# Benzidine and its salts: MAK Value Documentation – Translation of the German version from 2024

**DOI:** 10.34865/mb9287e11_2or

**Published:** 2026-06-30

**Authors:** Andrea Hartwig, MAK Commission

**Affiliations:** 1 Institute of Applied Biosciences, Department of Food Chemistry and Toxicology, Karlsruhe Institute of Technology (KIT) Adenauerring 20a, Building 50.41 76131 Karlsruhe Germany; 2 Permanent Senate Commission for the Investigation of Health Hazards of Chemical Compounds in the Work Area, Deutsche Forschungsgemeinschaft, Kennedyallee 40, 53175 Bonn, Germany. Further information: Permanent Senate Commission for the Investigation of Health Hazards of Chemical Compounds in the Work Area | DFG

**Keywords:** benzidine and its salts, human carcinogen, bladder cancer, carcinogenicity, germ cell mutagenicity, skin absorption, mechanism of action

## Abstract

The production and technical use of benzidine [92-87-5] and its salts are prohibited by law in the EU and many other countries. Although benzidine was added to the List of MAK and BAT Values in 1966 and classified in Carcinogen Category 1 in 1975, documentation for the substance had not yet been compiled. To remedy this, the German Senate Commission for the Investigation of Health Hazards of Chemical Compounds in the Work Area (MAK Commission) has now prepared the documentation and additionally evaluated the end points germ cell mutagenicity, sensitization and skin absorption. Relevant studies and reviews were identified from a literature search. The carcinogenicity of benzidine has not been re-evaluated in detail because there is unequivocal evidence of its carcinogenic potential in humans: Numerous case reports and epidemiological studies from different countries show a strong and consistent association between benzidine exposure and the risk of bladder cancer. Many studies found benzidine to be genotoxic in vitro and in vivo. No studies with germ cells are available. Findings that benzidine dihydrochloride crosses the blood-brain barrier in mice after oral administration suggest that the substance may also be able to pass through the blood-testis barrier. Therefore, benzidine and its salts have been classified in Category 3 A for germ cell mutagens. Benzidine is readily absorbed by rats after dermal application in acetone. The substance is a genotoxic carcinogen and a systemically tolerable dose cannot be derived. Therefore, the designation with "H" (for substances which can be absorbed in toxicologically relevant amounts) has been retained. No studies that investigated benzidine salts are available. Although the salts are probably absorbed less readily, absorption cannot be ruled out and the salts of benzidine also remain designated with "H". There are no studies that investigated the sensitizing effects of benzidine in animals. Studies using alternative test methods are also not available. A limited number of clinical findings show positive reactions to benzidine. As patients with sensitization to aromatic para-amino compounds, such as p-phenylenediamine, often react to other compounds of this substance class as well, cross-reactivity to benzidine is possible. The data available are too limited to draw a conclusion regarding the skin sensitizing potential of benzidine and its salts. Data for respiratory sensitization are not available.

**Table:** 

**MAK value**	**–**
**Peak limitation**	**–**
	
**Absorption through the skin (1966)**	**H**
**Sensitization**	**–**
**Carcinogenicity (1975)**	**Category 1**
**Prenatal toxicity**	**–**
**Germ cell mutagenicity (2023)**	**Category 3 A**
	
**EKA**	**not established**
	
Synonyms	4,4′-bianiline (1,1′-biphenyl)-4,4′-diamine 4,4′-diaminodiphenyl 4,4′-diphenylenediamine
Chemical name (IUPAC)	4-(4-aminophenyl)aniline
CAS number	92-87-5
Structural formula	Structural formula of benzidine. 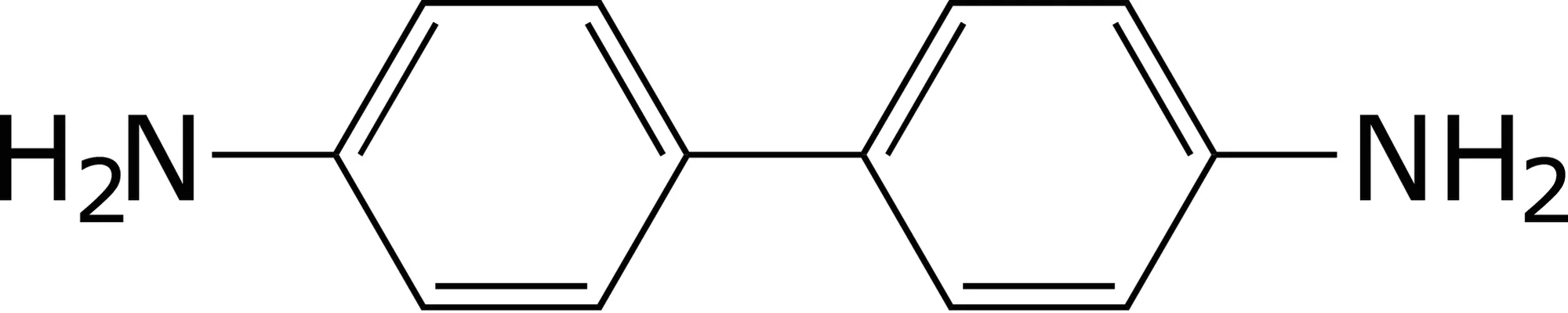
Molecular formula	C_12_H_12_N_2_
Molar mass	184.24 g/mol
Melting point	127–128 °C (Lewalter et al. 2016); 120 °C (NTP 2021)
Boiling point at 987 hPa	401 °C (Lewalter et al. 2016; NTP 2021)
Density at 20 °C	1.25 g/cm^3^ (Lewalter et al. 2016; NTP 2021)
Vapour pressure	6.67 × 10^–4^ hPa at 20 °C (Lewalter et al. 2016); 11.97 × 10^–7^ hPa at 25 °C (NTP 2021)
log K_OW_	1.34 (Lewalter et al. 2016; NTP 2021)
Solubility	322 mg/l water (NTP 2021)
	
Hydrolytic stability	no data
Bans on use and uses	The manufacture and technical use of benzidine and its salts are prohibited by law in the EU and many other countries (e.g. Japan, Canada and Switzerland). In the USA, benzidine has not been produced on a large scale since 1976, although small quantities are still available for diagnostic tests. It is produced and/or supplied in small quantities for research in Germany, the Hong Kong Special Administrative Region, India, the People’s Republic of China, Switzerland and the USA. The manufacture and use of benzidine in dye production is reported to take place in some developing countries, as is the relocation of benzidine production from other European countries to Serbia and Montenegro (IARC 2012).

Since 1994, the use of certain azo dyes in consumer goods that come into direct and prolonged contact with human skin (for example clothing, bed linen, shoes, gloves, etc.) has been prohibited in Germany. The dyes concerned are those which, following the reduction of one or more azo groups, can release one or more of 22 specific aromatic amines (including benzidine) in detectable concentrations (that is > 30 mg/kg). In 2002, this ban was imposed throughout the EU and affected also products that come into contact with the oral cavity. The FDA (US Food and Drug Administration) limited the benzidine content in food colourings to 1 µg/kg (IARC 2012). The aforementioned EU-wide ban has been regulated in Annex XVII (Point 43) of the REACH Regulation since mid-2009. According to the REACH Regulation, tattoo inks may contain a maximum of 5 µg benzidine/kg (European Parliament and European Council 2006).

For benzidine and its salts, documentation for the BAT value (Lewalter et al. 2016) and an addendum to the BAT value (Nasterlack 2016) are available to date.

Although benzidine was added to the List of MAK and BAT Values in 1966 and classified in Carcinogen Category 1 in 1975, documentation for the substance had not yet been compiled. To remedy this, this documentation has been prepared. A re-evaluation of the carcinogenicity of benzidine has not been carried out, as its carcinogenicity has been demonstrated in many studies. The end points germ cell mutagenicity, absorption through the skin and sensitization have been evaluated for the first time in this documentation.

This documentation is based mainly on reviews (ATSDR 2001, 2009; Carreón et al. 2006; IARC 2012; Lewalter et al. 2016; Nasterlack 2016; NTP 1980, 2021) and summaries of the end points relevant for assessment.

Like 2-naphthylamine and 4-aminobiphenyl, the aromatic amine benzidine is a proven human carcinogen. Its carcinogenicity, in the form of ‘urinary bladder cancer’, was recognized in aniline workers as early as 1900, but was misinterpreted for decades. The controversial discussions about the cause of increased incidences of bladder tumours in workers exposed to aminoaromatics could not be clarified for a long time. Even when in 1927 the bladder tumours in employees of a dyestuff production plant were clearly attributed to handling benzidine, these observations were still interpreted as the consequences of typical exposures to a mixture of substances. This assessment was not decisively influenced by the results of numerous subsequent studies, especially since animal experiments failed to prove the carcinogenicity of benzidine in mice and rats. Even the bladder tumours shown in 1950 in dogs exposed to benzidine led only to an improvement in the hygiene conditions in production, but not to a general ban on the production and handling of benzidine. The improved hygiene conditions could not reduce the morbidity rates for bladder tumours in the short term due to the long latency period of several decades from exposure to the manifestation of the effect. Benzidine production was not discontinued in England until the year 1962 and then in Germany and other western countries in 1967. Worldwide, but primarily in the Far East, 10 000 tonnes of all diphenyl bases, including benzidine, were still being produced in 1972 and 15 000 tonnes in 1983. The use of benzidine was not drastically restricted until after 1978, when the bioavailability of benzidine was demonstrated in metabolic studies of various benzidine dyes, initially in animal experiments and later also in the urine of workers (Lewalter et al. 2016).

## 1 Toxic Effects and Mode of Action

Benzidine is rapidly absorbed by inhalation and especially after dermal exposure. It is excreted via the kidneys in the form of the free or conjugated amine and conjugated metabolites with a half-life of up to 7 hours.

The carcinogenicity of benzidine in humans is proven. Tumours of the urinary tract are generally observed after long-term exposure to benzidine with latency periods of 20 years.

Numerous studies demonstrated the genotoxic effect of benzidine in vitro and in vivo in the form of mutations, clastogenicity and aneugenicity. No studies with germ cells are available. Findings that benzidine dihydrochloride crosses the blood–brain barrier in mice after oral administration suggest that the substance may also be able to pass the blood–testis barrier. It can therefore be assumed that benzidine or its metabolites reach the germ cells.

Only few studies are available for the sensitizing potential of benzidine. The clinical findings show that positive reactions to benzidine can occur. As patients with existing sensitization to aromatic para-amino compounds, such as *p*-phenylenediamine, often react also to other compounds of this substance class, cross-reactivity to benzidine is possible.

## 2 Mechanism of Action

### Development of bladder tumours

Benzidine is excreted via the kidneys in the form of the free or conjugated amine and conjugated metabolites. Strongly electrophilic intermediates are formed in the acidic urine, which leads to the formation of DNA adducts in the cells of the urothelium and is regarded as the tumour–initiating effect of benzidine in the bladder (Carreón et al. 2006, 2007).

#### Detoxification and influence of the acetylator status

**Fig. 1:**
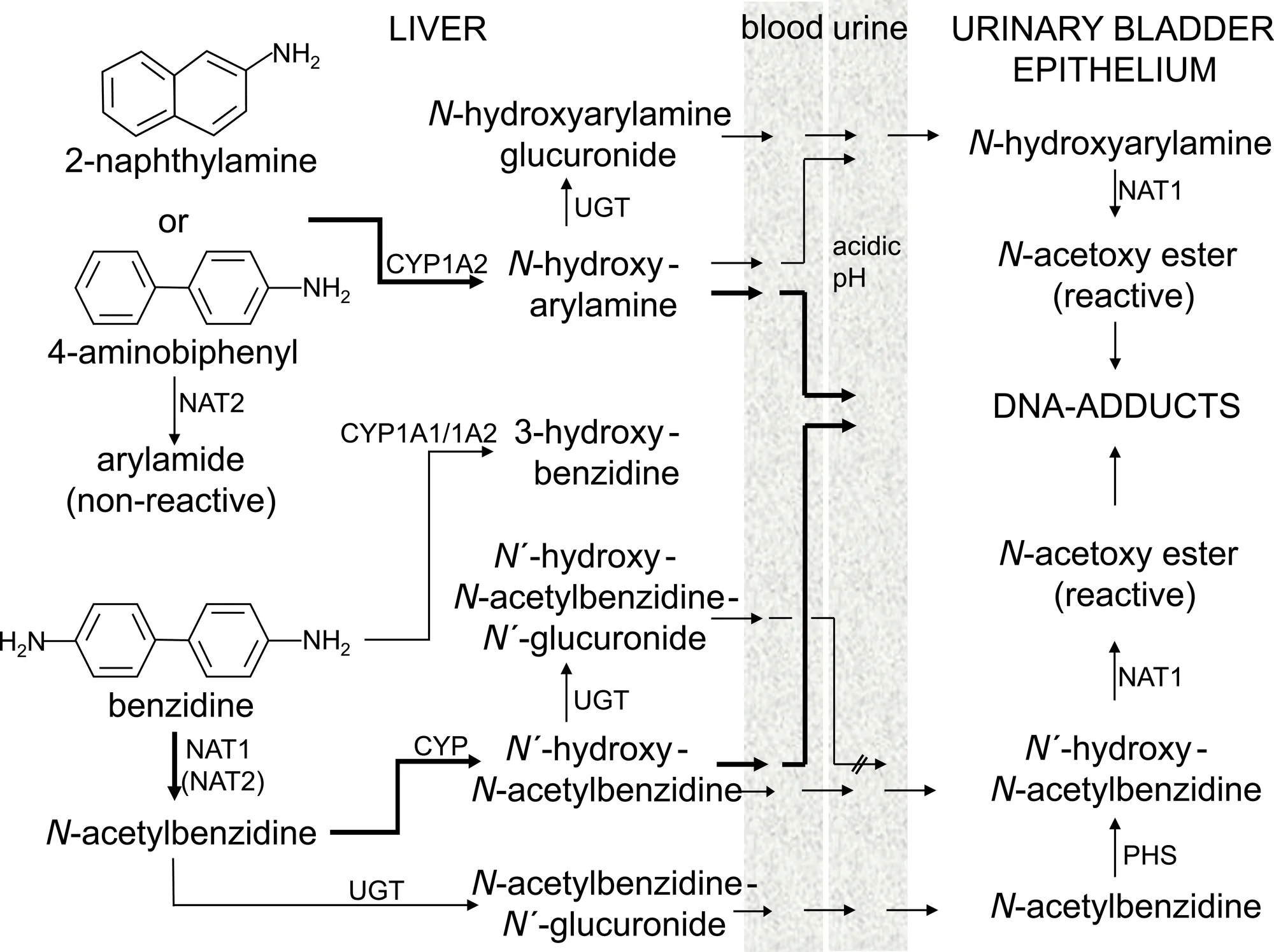
Metabolism of benzidine in comparison with other carcinogenic arylamines (according to Carreón et al. 2007, reproduced with permission of John Wiley & Sons, Inc., © 2007 Wiley-Liss, Inc.)

Studies of the metabolism of benzidine show that benzidine, which belongs to the diarylamines, is metabolized differently from the aromatic monoarylamines (such as 2-napthylamine and 4-aminobiphenyl). In contrast to the metabolism of monoarylamines, acetylation by *N*-acetyltransferase (NAT1 and NAT2) does not appear to be a detoxification step for benzidine. *NAT2* is polymorphic: a lack of two fully functional alleles leads to reduced NAT enzyme activity in affected individuals, who are slow acetylators. When exposed to monoarylamines, slow acetylators produce higher amounts of *N*-hydroxyarylamines than fast acetylators. *N*-hydroxyarylamines are considered to be the initiators of the carcinogenic process. Slow acetylators, on the other hand, do not have an increased risk of bladder cancer when exposed to diarylamines such as benzidine (Carreón et al. 2006). In the case of benzidine, the initial metabolites formed by *N*-acetylation at one amino group via NAT and subsequent oxidative metabolism at the second amino group lead to the formation of reactive metabolites with electrophilic properties which are held responsible for the formation of DNA and haemoglobin adducts and the resulting bladder tumours (Carreón et al. 2006; Pesch et al. 2013). The exclusively oxidative metabolism of both amino groups by cytochrome P450 enzymes (CYP) leads to detoxification (see Figure 1). Thus, *N*-acetylation is not a detoxification mechanism for benzidine, but presumably an important activation step. Both benzidine and *N*-acetylbenzidine are filtered into the bladder lumen after hepatic glucuronidation or hydroxylation and in the urine are released from their glucuronides due to the acidic pH. Other enzyme systems may contribute to the carcinogenicity of *N*-acetylbenzidine, too. It is possible that *N*′-hydroxy-*N*-acetylbenzidine is converted by NAT1 into *N*′-acetoxy ester, which can likewise contribute to the development of cancer in the bladder (Carreón et al. 2006, 2007).

Based on these data and the available epidemiological studies, it is therefore assumed that slow *N*-acetylation is associated with a lower risk of benzidine–induced bladder cancer. However, in vitro experiments have shown that both benzidine and *N*-acetylbenzidine are favoured substrates for NAT1, but not for NAT2. Thus, low NAT2 activity (slow acetylation) should not affect benzidine acetylation and the associated bladder cancer incidence. This is supported by a study in Indian employees in which the urothelial adducts were *N*-acetylated but not associated with NAT2 activity, NAT2 phenotype or NAT2 genotype. An increased but not statistically significant risk of developing bladder cancer was observed in those individuals who were homozygous or heterozygous for NAT1*10 (Carreón et al. 2006; Pesch et al. 2013).

**Summary:** In contrast to the metabolism of monoarylamines, acetylation by NAT is not a detoxification step for benzidine. In the case of benzidine, the initial metabolites formed by N-acetylation at one amino group via NAT and subsequent oxidative metabolism at the second amino group lead to the formation of reactive metabolites which are held responsible for bladder tumours. The data suggest that both benzidine and *N*-acetylbenzidine are favoured substrates for NAT1, but not for NAT2. Thus, low NAT2 activity (slow acetylation) should not affect benzidine acetylation and the associated bladder cancer incidence.

## 3 Toxicokinetics and Metabolism

Benzidine is rapidly absorbed by inhalation and especially after dermal exposure, and is excreted via the kidneys in the form of the free or conjugated amine and conjugated metabolites with half-lives of up to 7 hours (ATSDR 2001; Lewalter et al. 2016).

Dermal absorption was investigated in male F344 rats (225–250 g body weight). ^14^C-Labelled benzidine (purity not specified; 1 mg/kg body weight, dissolved in 0.2 ml acetone) was applied to 5–6 cm^2^ of shaved dorsal skin. After 60 minutes, 8 and 24 hours, the radioactivity in the organs, carcass, urine and faeces was determined. At the three time points, 94.6%, 75.1% and 48.6%, respectively, remained at the application site. After 1 hour, a total of about 4.4% of the applied radioactivity was found in the liver, intestine and carcass; after 8 hours about 4.1% was recovered in the urine and about 0.7% in the faeces, after 24 hours 22.8% and 18.7%, respectively (Shah and Guthrie 1983). From the data obtained after 1 hour a flux of 1 mg/kg body weight / 0.238 kg body weight / 5.5 cm^2^ × 0.043 = 32.8 µg/cm^2^ and hour can be calculated.

The metabolism of diarylamines, such as benzidine, is different from that of monoarylamines. In contrast to aromatic amines, benzidine is a good substrate for cytochrome P450 only after *N*-acetylation (Leng et al. 2019). Benzidine is therefore first acetylated in the liver to *N*-acetylbenzidine and then, unlike monoarylamines, has another free amino group that is accessible for *N*′-oxidation to *N*′-hydroxy-*N*-acetylbenzidine or for *N*′-glucuronidation to *N*-acetylbenzidine-*N*′-glucuronide. The free amino group can be further acetylated to *N*,*N*′-diacetylbenzidine by NAT. The products of hepatic glucuronidation or hydroxylation are filtered into the bladder, where they can be degraded by the acidic urine (Carreón et al. 2006, 2007; Section 2; Figure 1).

## 4 Effects in Humans

This section lists only studies investigating the relevant end points sensitization, genotoxicity and carcinogenicity.

### 4.1 Allergenic effects

There are only few studies available for the sensitization potential of benzidine as exposure is rare due to its carcinogenic effects in humans.

#### 4.1.1 General studies

Of 100 patients who yielded positive results for *p*-phenylenediamine, benzidine and benzocaine, a total of 11 reacted to only 1 of the aromatic amines tested in a second series of tests: 4 of them to benzidine (concentration not specified), 4 and 3 to *p*-phenylenediamine and benzocaine (no other details), respectively. Of the remaining 89 patients who reacted to at least 2 aromatic amines, 2 exhibited an identical sensitization pattern: the test results were positive for benzidine, *p*-toluidine and *p*-aminophenol, but negative for a further 29 aromatic amines, azobenzene and 2 azo dyes (Rudzki 1975). The reason for testing with benzidine was not explained. No information is available for the test concentration used, the reading times, the time course and the reaction strength. Therefore, this study is not used for the evaluation.

Of 76 patients with atopic dermatitis, 1.3% (n = 1) reacted to 1% benzidine in petrolatum; in 895 patients with other forms of dermatitis (for example nummular eczema), the percentage of positive reactions was 8.1% (Rudzki and Grzywa 1975). Readings were taken 48 and 96 hours after application (Rudzki and Kleniewska 1970). This study likewise provides no information on the time course and strength of the reactions.

#### 4.1.2 Studies with occupational exposure

Of 4600 patients referred to a dermatological clinic in Spain in the period from 1973 to 1977, 927 (20.2%) reacted in the patch test to the ‘para-compounds’ tested (including benzidine, *p*-phenylenediamine, *p*-aminophenol, sulphonamides and the local anaesthetics procaine and benzocaine). Of the 927, 231 produced positive reactions to 3% benzidine in petrolatum (5% of the total collective), of which 153 (66%) reacted only to benzidine and 78 (34%) to both benzidine and other allergens. In 38 (16.4%) a cross-reaction within the group of para-compounds tested was suspected. According to the authors, occupational allergic contact dermatitis was clinically diagnosed in 208 of the 231 patients (66 workers in the metallurgical and engineering industries, 55 housewives (contact by washing and rinsing dyed cotton textiles), 41 construction workers, 20 workers in the textile industry, 14 hairdressers and 11 workers in the chemical industry). However, the only common factor between them appears to be the wearing of clothing or footwear coloured with benzidine–based dyes. According to the authors, the use of these dyes had decreased in Spain at the time of the study, but had not yet been discontinued (Grimalt and Romaguera 1981; Romaguera and Grimalt 1980). There is no information available for the reading times, the time course and the reaction strength. The workplace relevance of the posi­tive reactions appears questionable. Furthermore, cross-reactivity with other aromatic para-amino compounds such as *p*-phenylenediamine is possible.

In a cross-sectional study conducted between March and December 2009 with a total of 472 employees from 2 tanneries in Indonesia, 77 people with current dermatitis were identified using questionnaire interviews and skin findings. Patch tests were performed on 63 of these 77 employees and on 108 tannery employees without skin disease as controls. The readings were taken on days 2, 4 and 7. Three of the 63 persons tested (3.9%) reacted in the patch test with 1% benzidine in petrolatum, 13 with current workplace-related contact dermatitis produced reactions to one or more of the 15 allergens tested. In only one of the 3 employees with a reaction to benzidine was a benzidine-based dye found in the tannery’s list of chemicals, which the authors described as a current relevant exposure. For the other two, previous exposure is suspected (Febriana et al. 2012 a, b, c). There is no information available for the reaction strength and the course of the reaction. Testing with the benzidine-based dye used in the tannery was evidently not carried out. It is not clear whether the positive reactions of the 3 employees were due to exposure to a benzidine-based dye or to its decomposition on the skin and the resulting release of benzidine components.

#### 4.1.3 Case reports

The following individual case report describes the development of allergic dermatitis directly related to occupational exposure to benzidine. A 32-year-old physician with severe, recurrent eczematous dermatitis on his hands and to a lesser extent on his face produced a strong reaction in the patch test with benzidine (as a solid). The dermatitis always developed after the benzidine test was performed to detect blood in the faeces of patients. Patch tests with other materials with which the physician came into professional contact were negative (Baer 1945).

### 4.2 Genotoxicity

A study investigating cytogenetic effects in 23 workers who had been exposed to benzidine (0.42–0.86 mg/m^3^) and benzidine-based dyes (7.8–32.3 mg/m^3^) for an average of 15 years revealed a statistically significant increase in peripheral lymphocytes with chromosomal aberrations compared with the control values (ATSDR 2001).

### 4.3 Carcinogenicity

The carcinogenicity of benzidine in the human bladder is proven. It is characterized by broad-based or pedunculated papillomas that degenerate into cancer. Also primary transitional cell carcinomas were observed. Numerous case reports and studies from various countries have been published on bladder tumours in humans after exposure to benzidine and have been discussed and evaluated by various authors and committees. Overall, the case reports and epidemiological studies show a strong and consistent association between benzidine exposure and the increased risk of bladder cancer in humans (ATSDR 2001, 2009; IARC 2012; Lewalter et al. 2016; Nasterlack 2016; NTP 1980, 2021).

The following are the most important study results in key points from the IARC (2012):

- all 5 of a group of workers who had been employed in benzidine production for at least 15 years developed bladder cancer
- in 20 of 83 Italian workers involved in the production and use of benzidine in the dye industry, bladder tumours were diagnosed in the period from 1931 to 1948
- 10 deaths from bladder cancer in dye industry workers exposed only to benzidine (no other details; standardized mortality ratio (SMR) 13.9; 95% CI: 6.7–25.5)
- in workers exposed to benzidine in the dye industry in China, morbidity from bladder cancer increased with the increasing duration of exposure (p for trend < 0.01)
- in a cohort of benzidine manufacturing workers in the USA, the risk of dying from bladder cancer was significantly increased for those with at least 2 years of exposure to benzidine (SMR 13.0; 95% CI: 4.8–28.4)
- in a cohort of workers in the dye industry in Turin, Italy, the SMR was 100.8 during exposure and 14.8 twenty or more years after exposure ceased
- among workers at a chemical manufacturing plant in Shanghai, China, the relative risk of bladder cancer after exposure to benzidine was 63.4 (p < 0.05) for non-smokers compared with that of unexposed non-smokers; for smokers without exposure the relative risk was 6.2 (p = 0.05) and with exposure 152.3 (p < 0.01)
- among Chinese workers in the production and use of benzidine, the odds ratios for bladder cancer were 1.0, 2.7 (1.1–6.3) and 4.4 (1.8–10.8) for low, medium and high cumulative exposure to benzidine, respectively, after adjustment for lifetime cigarette smoking
- in a case–control study in Canada, excesses of renal cell cancer in relation to the duration of exposure to benzidine (p < 0.004) were noted

The following studies have been published following the IARC assessment (2012):

A 71-year-old former worker who had worked in cotton dyeing factories for 29 years, including 3 years as a dyer, suffered from cancer of the upper urinary tract. Mainly benzidine-based dye was used. The benzidine concentration was in the range from below the detection limit up to 397.4 μg/m^3^. The cancer was diagnosed in 2018 with a latency period of about 35 years. The authors consider benzidine exposure to be a probable cause of cancer not only in the bladder but also in the upper urinary tract (Kim et al. 2020).

In a cohort of workers (n = 488) who worked at the last benzidine production plant in the USA, a follow-up study conducted up to 2014 continued to show an increased risk of developing bladder cancer. At the time of the study, at least 41 years had passed since the workers were exposed. Both incidence and mortality were increased with statistical significance (25 new cases, standardized incidence ratio (SIR) 2.19 (95% CI: 1.42–3.23) and 5 deaths, SMR 3.79 (95% CI: 1.23–8.84)). There was a significant increase in incidence and mortality in those exposed to both benzidine and dichlorobenzidine (SIR 3.11 (95% CI: 1.97–4.67), SMR 4.10 (95% CI: 1.12–10.5)), but not in workers exposed only to dichlorobenzidine (2 new cases of disease, SIR 0.89 (95% CI: 0.11–3.23) and 1 death, SMR 2.90 (95% CI: 0.07–16.15)). The incidence of bladder cancer and resulting mortality were increased in workers with more than 5 years of employment and a latency period of more than 20 years since the last exposure (6 observed, SIR 5.94 (95% CI: 2.18–12.92) and 2 deaths, SMR 7.93 (95% CI: 0.96–28.65)) (Millerick-May et al. 2021).

Studies with unclear exposure or co-exposure to other substances are not listed.

## 5 Animal Experiments and in vitro Studies

This section lists only studies of the relevant end points sensitization, genotoxicity and carcinogenicity.

### 5.1 Allergenic effects

There are no data available.

### 5.2 Genotoxicity

There are numerous in vitro and in vivo studies that demonstrate the genotoxicity of benzidine. The substance causes mutations in bacteria and genotoxic effects in various test systems in vitro in human and mammalian cells. In rodents, benzidine induced micronuclei, DNA strand breaks, unscheduled DNA synthesis, chromosomal aberration, sister chromatid exchange and aneuploidy (for details see ATSDR 2001, 2009). In pregnant mice, increased incidences of micronuclei were found in the liver of the foetuses after intraperitoneal administration of benzidine, suggesting that benzidine or its metabolites cross the placental barrier (ATSDR 2001).

There are neither more recent studies available nor studies with germ cells. In a study with lifelong administration of benzidine dihydrochloride via the drinking water, effects occurred in the brain of mice (spongiform leukoencephalop­athy) (Littlefield et al. 1983; Morgan et al. 1981). This demonstrates that the substance is able to cross the blood–brain barrier. Based on these data, it can likewise be assumed that benzidine and its salts pass the blood–testis barrier and thus reach the germ cells.

### 5.3 Carcinogenicity

There is sufficient evidence for the carcinogenicity of benzidine in animals. Oral administration of benzidine led to increased tumour incidences in the mammary gland of female rats, in the liver of mice and hamsters and in the bladder of dogs. After subcutaneous administration, benzidine caused Zymbal gland tumours in rats and liver tumours in mice, and after intraperitoneal injection, Zymbal and mammary gland tumours in rats (IARC 1982, 1987; NTP 2021).

## 6 Manifesto (MAK value/classification)

**Carcinogenicity. **The carcinogenicity of benzidine in the human bladder is proven. It is characterized by broad-based or pedunculated papillomas that degenerate into cancer. Also primary transitional cell carcinomas are observed. Numerous case reports and epidemiological studies from various countries have shown there to be a strong and consistent association between benzidine exposure and the risk of bladder cancer in humans. Benzidine was therefore classified in Carcinogen Category 1. This classification has been retained.

**Germ cell mutagenicity. **Numerous studies found benzidine to be genotoxic in vitro and in vivo as manifested in the form of mutations, clastogenicity and aneugenicity. No studies with germ cells are available. Findings that benzidine dihydrochloride crosses the blood–brain barrier in mice after oral administration suggest that the substance may also be able to pass the blood–testis barrier (Li et al. 2016; Wen et al. 2018). It must therefore be assumed that benzidine or its metabolites reach the germ cells. Therefore, benzidine and its salts are classified in Category 3 A for germ cell mutagens.

**Absorption through the skin. **Benzidine is readily absorbed by rats after dermal application in acetone. The substance is a genotoxic carcinogen and a systemically tolerable dose cannot be derived. Therefore, designation with an “H” (for substances which can be absorbed in toxicologically relevant amounts) has been retained. Studies with benzidine salts are not available. Although the salts are probably absorbed less readily, absorption cannot be ruled out and the salts of benzidine also remain designated with an “H”.

**Sensitization. **There are no studies available for the sensitizing effects of benzidine in animals. Studies using alternative test methods are likewise not available. Clinical findings show that positive reactions to benzidine do occasionally occur. A contact sensitizing effect of benzidine itself in humans cannot be deduced from the available clinical data due to the methodological shortcomings of the mostly older studies. As patients with sensitization to aromatic amino compounds, such as *p*-phenylenediamine or aniline, often react to other compounds of this substance class as well, cross-reactivity to benzidine cannot be excluded. In the light of the available data, benzidine has not been designated with “Sh” (for substances which cause sensitization of the skin). Data for respiratory sensitization are not available. Benzidine and its salts are therefore not designated with “Sa” (for substances which cause sensitization of the airways).

**MAK value, peak limitation, pregnancy risk group **A MAK value has not been established. Therefore, peak limitation and the assignment to a pregnancy risk group are not applicable.
